# Case report: a unique presentation of memantine overdose causing echolalia and hypertension

**DOI:** 10.1186/s12877-024-04658-2

**Published:** 2024-02-01

**Authors:** Sana Durrani, Shaista Ahmed

**Affiliations:** Division of Geriatrics and Gerontology, Johns Hopkins School of Medicine, 5200 Eastern Avenue, Mason F Lord Building, Center Tower, Suite 2200, 21224 Baltimore, MD USA

**Keywords:** Memantine, Echolalia, Hypertensive crisis, Adverse effects, Dementia

## Abstract

**Background:**

Since 2003 when memantine was first approved for use in the management of moderate-severe Alzheimer’s dementia, its use has become more widespread and is being explored in other diseases like neuropathic pain, epilepsy, and mood disorders. Our case uniquely highlights two important adverse effects in a patient who overdosed on memantine. One is hypertension, which is easy to overlook as a medication side effect. The other is echolalia which is the repetition of words and phrases spoken by another person. It is commonly seen in children with autism spectrum disorder and has been reported in older adults with head injuries, delirium, and neurocognitive disorders. The aim of this patient story is to highlight the importance of medication reconciliation with caregivers and knowledge of adverse drug reactions in patient management. This case report has been presented previously in the form of an abstract at the American Geriatrics Society Presidential poster session in May 2023.

**Case presentation:**

Our patient is an 86-year-old man with mild dementia and hypertension, who was brought to the emergency department (ED) due to abrupt onset of altered mental status and auditory hallucinations. Investigations including blood work, CT head and an electroencephalogram (EEG) did not reveal an etiology for this change in his condition. Due to elevated blood pressure on presentation, a nicardipine drip was started, and he was given IV midazolam to assist with obtaining imaging. While reviewing medications with his daughter, it was noted that sixty memantine pills were missing from the bottle. Poison control was contacted and they confirmed association of these features with memantine. With supportive care, his symptoms resolved in less than 100 h, consistent with the half-life of memantine. Notably, our patient was started on Memantine one month prior to this presentation.

**Conclusions:**

Hypertensive urgency and echolalia were the most striking symptoms of our patient’s presentation. Though hypertension is a known sign of memantine overdose, it can easily be contributed to medication non-compliance in patients with dementia, being treated for hypertension. According to our literature review, this the first case of memantine overdose presenting with echolalia, a sign that is not commonly associated with adverse reactions to medications. This highlights the importance of an early medication review, especially with caregivers of people with dementia.

## Background

In 2020, nearly 4 million prescriptions for memantine were written, making it the 152nd most prescribed medication in the United States at that time and an increasingly prescribed medication worldwide [1–4]. Memantine is an N-methyl-D-aspartate (NMDA) receptor antagonist that prevents overactivation of the glutamate system while allowing physiologic activity [5]. It also exhibits antagonist activity at the serotonergic and acetylcholine receptors and reaches a maximum concentration in the body between 3 and 8 h with an elimination half-life of between 60 and 100 h. [5–6]. Its use has become widespread partly because unlike other NMDA receptor antagonists, it is reportedly well tolerated with few deleterious side effects [5]. This has been reported to be due to differences in the timing of drug elimination, in transitions between blocked states of the receptor, and in the number of bound sites on NMDA receptors [5]. Dizziness, confusion, and headaches are some of its most reported adverse effects [6]. Our case uniquely highlights two important adverse effects in a patient who overused memantine. One is hypertension, which is commonly managed and easy to overlook as a medication side effect. It presented as hypertensive crisis in our patient. The other is echolalia, or meaningless repetitive speech. This is a sign that is usually seen in behavioural disorders or strokes, not medication overdose. To our knowledge, echolalia has not previously been published as an adverse effect of regular or overdose of this medication (Table 1).

## Case presentation

Our patient is an 86-year-old man who was brought to the ED by emergency medical services due to behavioural concerns reported by his daughter. These included the abrupt onset of altered mental status with auditory hallucinations, repetition of words and phrases, and intermittent episodes of agitation followed by periods of somnolence. His comorbidities included Alzheimer’s dementia that had been diagnosed one year prior to this presentation, hypertension, hyperlipidaemia, osteoarthritis, syringomyelia post laminectomy, and peripheral arterial disease. Notable home medications included memantine, which had been started 1 month prior to this presentation at a dose of 5 mg twice a day, metoprolol succinate 50 mg daily, and meclizine 12.5 mg every 8 h as needed for chronic dizziness.

On presentation in the ED, his blood pressure was 197/79, heart rate 97, respiratory rate 15, and oxygen saturation above 95% on room air. There were no significant abnormalities in laboratory testing which included a complete blood count, comprehensive metabolic panel, lipase level, ethanol level and drug toxicology. His electrocardiogram showed a normal sinus rhythm with a few premature ventricular contractions and a normal QTc interval. CT head without contrast showed chronic microvascular changes and age-related diffuse parenchymal volume loss. On physical exam, he was alert, only oriented to self and experiencing auditory hallucinations. He would repeat questions exactly as they were said to him. There was no nystagmus, tremor, or rigidity noted on exam. Due to concerns for respiratory compromise he was observed in the Intensive Care Unit. An urgent EEG was performed and did not show epileptiform activity. Neurology and psychiatry were also consulted to help determine the etiology of his waxing and waning mental status and altered behaviours.

While gathering collateral information from his daughter, medication review and compliance was discussed. It was discovered that there were at least 60 missing pills from memantine pill bottle. The provider contacted poison control, who confirmed that the hypertensive crisis, behavioural disturbances, and repetitive precise speech could all be attributed to memantine overuse.

After 96 h all his symptoms had resolved. He returned to his baseline cognition. His initial MMSE score which was 13/25 had improved to 21/28 prior to hospital discharge. Our patient had no recollection of the events leading to this hospitalization. His family felt that he may have mistaken the memantine bottle for meclizine bottle given the similarity in names and may have been trying to use additional memantine as his dizziness had worsened recently. Him and his family also reported increased anxiety over the last few months with mild subjective worsening of cognitive functioning. Escitalopram was added for anxiety prior to discharge and memantine was discontinued. The daughter later confirmed that the patient had mixed up Memantine with Meclizine and had been overusing Memantine over the prior week. We discussed medication supervision and compliance with him and his family in detail. We reviewed our patient’s records approximately one year after the hospital stay at the time of submission of this report, and he continues to live at home with the support assistance of his family and 24-hour caregivers.

## Discussion

The combination of behavioral disturbance, hypertension, and hallucinations in a patient with dementia led us to consider a wide variety of etiologies, including cerebrovascular accidents, infectious etiologies, metabolic abnormalities, intoxication, and delirium. Specifically, when evaluating his echolalia, we noted that echolalia has been reported in certain types of dementia and strokes, and in delirious states [8]. Due to acuity of his symptoms, this was not considered to be directly related to his dementia and our work up did not support metabolic, infectious, structural or vascular causes for encephalopathy. Discussion with family and their engagement in the evaluation of his complex medical presentation was the key to diagnosis and appropriate management. This serves as a reminder for all clinical providers of the value of medication review and gathering information from caregivers.

Memantine has been found to have a favorable tolerability profile when used as monotherapy or in combination with other agents. Serious adverse effects are not common. Most reported adverse effects include headaches, bowel habit changes, dizziness, and confusion [6]. A 2019 Cochrane review discussed these effects, reporting that memantine was probably 1.6 times more likely to cause dizziness and 1.3 times more likely to cause headaches than placebo [7]. Hypertension was also noted to be a common side effect in a review article on Memantine’s safety and tolerability [8]. Its use is being studied extensively in multiple conditions apart from dementia, including mood disorders such as bipolar disorder and schizophrenia, epilepsy, neuropathic pain, and autism spectrum disorder [9–14]. The increasing frequency of its use, particularly in our vulnerable populations who may inadvertently take higher doses of this medication than prescribed, makes it important for providers and caregivers to have knowledge of the adverse effects of both regular and over usage of memantine. Our literature review revealed 3 published cases of memantine overdose and a few reported cases of memantine overdose, all with varying dosages and subsequent symptoms [15–17]. Their symptomatology is summarized in Table 1. None of these resulted in mortality.

**Table 1:** Summary of signs and symptoms from reported memantine overdose cases

CASES	1 2000 mg, acute	2 400 mg, acute	3 50 mg/day, > 12 months	4 105–200 mg/day, 3 days	5 Unknown doses
REPORTED SIGNS AND SYMPTOMS	Comatose Nystagmus, mydriasis, diplopia Disorientation Tachycardia, hypertension	Agitation Visual hallucinations Psychosis Somnolence, unconsciousness Proconvulsive state	Dyspepsia	Tiredness, weakness Diarrhea No symptoms	CNS (confusion, drowsiness, somnolence, vertigo, agitation, aggression, hallucination, and gait disturbance) Gastrointestinal (vomiting and diarrhea)

Our patient displayed many of these previously reported symptoms, including agitation, confusion, somnolence, and hallucinations. His symptoms also resolved within 100 h, consistent with the half-life of memantine [6]. Notably, our patient was also in hypertensive crisis, which improved with a Nicardipine drip started in the ED. Hypertension is a previously reported adverse effect of memantine but may not be quickly recognized as a medication adverse reaction when a patient presents in a hypertensive crisis. If the patient is given frequent anti-hypertensives, after the drug’s half-life has been reached, they may become hypotensive instead. The repetition of questions that were directed to him is significant, as it has not been previously published in relation to memantine use to our knowledge. Echolalia is common in young children and is an important component of language acquisition. It’s pathogenesis though not fully understood, has been linked to dopaminergic dysregulation and frontal lobe dysfunction [18]. Although it has been reported as a possible adverse effect of a few drugs, such as topiramate, baclofen, and ifosfamide, its differential is most commonly behavioral disorders or strokes [19–21]. A possible etiology of echolalia in the overuse of memantine can be related to the role of NMDA receptors in learning and memory processes. It has previously been suggested that because Memantine may module the glutaminergic processing at synpases, it may assist in aphasia in certain cases [22]. An overdose of Memantine therefore could potentially lead to non-physiologic neuronal activity levels resulting in echolalia. The complications of echolalia have mostly been studied in children and include impaired social interaction and learning, mood disorders and aggression. Treatment usually includes working with a team to find the etiology of echolalia and working with a speech language pathologist on specific therapies based on the etiology. Interestingly, memantine, in combination with aphasia therapy, has been used as a cognitive enhancing drug to improve mitigated echolalia in a patient with fluent aphasia [23]. Its use as a potent neuromodulator continues to be studied.

## Conclusion

We described a case of suspected memantine overdose that highlights the importance of collecting collateral information, performing a medication reconciliation and the role of poison control when dealing with uncommonly reported adverse effects of medications. Our patient helps remind us to be vigilant in recognizing hypertension as a drug adverse effect and exploring medication related causes for symptoms that are not commonly seen in older adults.

Increased awareness of these uncommon adverse effects may help providers and other caregivers recognize early signs of overdose and avoid unnecessary interventions and prolonged hospitalization. Recognition of these effects can also help facilitate discussions between providers, patients, around deprescription of the medication when appropriate.

## Data Availability

All patient data was obtained via the electronic medical record system at Johns Hopkins.

## Abbreviations

ED: Emergency Department
ECG: Electrocardiogram
EEG: Electroencephalogram
CT: Computerized topography
MRI: Magnetic resonance imaging
MMSE: Mini-mental status exam
NMDA: N-methyl-D-aspartate
